# Hybrid Alginate-Based Polysaccharide Aerogels Microparticles for Drug Delivery: Preparation, Characterization, and Performance Evaluation

**DOI:** 10.3390/gels11100775

**Published:** 2025-09-26

**Authors:** Mohammad Alnaief, Balsam Mohammad, Ibrahem Altarawneh, Dema Alkhatib, Zayed Al-Hamamre, Hadeia Mashaqbeh, Khalid Bani-Melhem, Rana Obeidat

**Affiliations:** 1 Pharmaceutical and Chemical Engineering Department, School of Applied Medical Sciences, German Jordanian University, Amman Madaba Street, P.O. Box 35247, Amman 11180, Jordan; 2 Department of Chemical Engineering, School of Engineering, The University of Jordan, Amman 11942, Jordan; 3 Pharmaceutics and Pharmaceutical Technology Department, Faculty of Pharmacy, Yarmouk University, Irbid 21163, Jordan; 4 Center for advanced Material (CAM), Qatar University, Doha P.O. Box 2713, Qatar; 5 Department of Pharmaceutics and Pharmaceutical Technology, Faculty of Pharmacy, The University of Jordan, Amman 11942, Jordan

**Keywords:** hybrid aerogels, polysaccharide, drug delivery, supercritical fluid extraction, microparticles

## Abstract

Hybrid polysaccharide-based aerogels offer significant potential as advanced drug delivery platforms due to their tunable structure, high porosity, and biocompatibility. In this study, aerogel microparticles were synthesized using alginate, pectin, carrageenan, and their hybrid formulations via an emulsion–gelation technique followed by supercritical fluid CO_2_ extraction. The resulting aerogels exhibit mesoporous structures with specific surface areas ranging from 324 to 521 m^2^/g and pore volumes between 1.99 and 3.75 cm^3^/g. Comprehensive characterization (SEM, gas sorption, XRD, TGA, DSC, and FTIR) confirmed that hybridization improved morphological uniformity and thermal stability compared to single polymer aerogels. Ibuprofen was used as a model drug to evaluate loading efficiency and release kinetics. Among all formulations, the alginate/carrageenan (2:1) hybrid showed the highest drug loading efficiency (93.5%) and a rapid release profile (>90% within 15 min), closely matching the performance of commercial ibuprofen tablets. Drug release followed Fickian diffusion, as confirmed by the Korsmeyer–Peppas model (R^2^ > 0.99). These results highlight the potential of hybrid polysaccharide aerogels as vehicles for drug delivery and other fast-acting therapeutic applications.

## 1. Introduction

Aerogels are increasingly considered a promising platform for drug delivery systems (DDS), particularly for oral administration, due to their unique physical properties [1,2,3,4,5]. These ultra-light materials are distinguished by their remarkable porosity, large surface area, and low density, making them ideal for encapsulating and delivering drugs in a controlled and sustained manner [2,6,7,8]. Polysaccharide-based aerogels have gained widespread interest because of their superior biological compatibility and mechanical properties, which are vital for pharmaceutical applications [9,10,11]. These materials can be synthesized by forming a hydrogel, followed by solvent exchange (often ethanol), and supercritical drying using CO_2_, which preserves their high porosity and low density [12,13]. Polysaccharide-based aerogels, such as those made from chitosan and alginate, offer distinct advantages for oral drug delivery. These materials are increasingly recognized as promising drug carriers due to their high loading capacities, making them suitable for various drug delivery applications [14,15,16]. Different polysaccharides, including alginate, pectin, and starch, have been processed using technologies like emulsification or drip-gelation to produce aerogel particles capable of incorporating drugs in their amorphous form. These formulations exhibit modified drug release profiles, offering significant potential for improving drug absorption and stability [17,18,19]. Additionally, hybrid polymers have emerged as promising drug carriers [20,21,22]. Blends of chitosan and alginate polymers have shown notable potential in controlled-release formulations [23,24] and in the preparation of micro and nanoparticles for drug delivery, particularly for protein-based therapies [25]. The drug-loading process can occur at various stages of synthesis, particularly during gel formation, solvent exchange, +or supercritical drying, and this flexibility allows for efficient incorporation of active pharmaceutical ingredients [26,27].

The use of biopolymer-based aerogels extends beyond their mechanical properties [28]. They offer several other beneficial characteristics, including a high surface area for drug encapsulation, the ability to stabilize drugs in an amorphous state to improve solubility, and the capacity to modulate drug release profiles [2,29]. These features are crucial for overcoming challenges such as poor drug solubility and instability, which are common issues faced by new molecular entities in drug development. In addition to enhancing solubility, the mesoporous structures of polysaccharide-based aerogels improve drug loading, providing significant potential for the delivery of poorly soluble drugs [29,30,31,32]. Polysaccharide-based aerogels are effective in drug delivery for a variety of applications, including wound healing and insulin delivery. For instance, Jia Xu et al. demonstrated that alginate-based aerogels could efficiently adsorb lysozyme, making them promising for skin wound management due to their high drug-loading capacity and controlled release profiles [33]. Similarly, Ozesme Taylan et al. developed core–shell alginate aerogels that improved insulin encapsulation and release, offering an alternative to conventional injection methods by improving bioavailability and providing sustained drug release in simulated gastric and intestinal fluids [1]. Gonçalves et al. further emphasized the importance of polysaccharide aerogels by creating hybrid alginate-based microparticles for mucosal drug delivery, with applications for ketoprofen and quercetin [34]. These aerogels exhibited high drug loading and controlled release, enhancing drug absorption through mucosal membranes. Additionally, Alnaief et al. explored chitosan-alginate aerogels for pulmonary delivery, where they demonstrated that the microparticles’ physicochemical properties could be tailored to optimize drug release and bioavailability [13].

Biodegradable polymers, including both natural and synthetic types, are often utilized in drug delivery systems due to their ability to enhance drug stability, improve targeting, and minimize side effects. Natural polysaccharides like chitosan and alginate, and synthetic polymers such as PLGA and PLA, offer various benefits, including controlled degradation and biocompatibility [9,35]. These polymers can degrade into simpler, non-toxic compounds within the body, which makes them particularly suitable for applications in areas such as dermatology, oral drug delivery, and wound healing. Their capability to fine-tune drug release profiles over extended periods enhances therapeutic outcomes while reducing the risk of unwanted side effects. Moreover, the versatility of aerogels extends to the formulation of hybrid systems, which combine polysaccharides with synthetic polymers to optimize drug delivery. These hybrid aerogels allow for better control of release kinetics, increased drug loadings, and improved drug bioavailability, particularly for poorly soluble compounds [9,35]. Such advancements are crucial in addressing challenges associated with the oral delivery of therapeutic agents, including insulin, and in enhancing the efficiency of drug delivery systems for a range of medical applications.

Ibuprofen is a widely used model drug, according to the Biopharmaceutics Classification System (BCS) it is a Class II compound, characterized by low aqueous solubility and high intestinal permeability, making it a standard benchmark for testing dissolution-enhancing formulations. In addition, ibuprofen is soluble in supercritical CO_2_ under the operating conditions used in this study, enabling its in situ incorporation into the aerogel network. While ibuprofen has previously been used as a model drug in polysaccharide aerogel systems, the novelty of this work lies in the systematic comparison of binary alginate–pectin and alginate–carrageenan hybrids with their respective single-polymer controls. By evaluating these systems under identical processing conditions, we were able to clearly isolate the synergistic contributions of hybridization to textural properties, loading efficiency, and release behavior. To the best of our knowledge, this is among the first works to demonstrate alginate’s role as a structural backbone that reinforces weaker polysaccharides, thereby outlining a design strategy for tailoring drug delivery performance in hybrid polysaccharide aerogels.

In the presented study, alginate, pectin, and carrageenan were selected due to their complementary functionalities and potential synergistic interactions. Alginate is well known for its ionic gelation through calcium ions, forming an egg-box structure, which provides strong networks with high porosity [36]. Pectin, with its variable degree of esterification, offers tunable crosslinking and mucoadhesive properties [37], while carrageenan enhances water uptake and can promote faster drug release through increased matrix hydration [12]. The hybridization of the selected polymer was hypothesized to yield improved structural uniformity, synergistic crosslinking, and enhanced drug loading/release performance compared to single-polymer systems [17,34]. The aerogel microparticles were prepared using an emulsion–gelation process followed by successive solvent exchange and CO_2_ supercritical drying. The aerogels were characterized using SEM, N_2_ gas sorption analysis, DSC, TGA, and FTIR. Selected samples were loaded with ibuprofen and further evaluated by XRD, FTIR, loading efficiency determination, and in vitro release studies.

## 2. Results and Discussion

### 2.1. Morphology and Textural Properties

**Figure 1:**
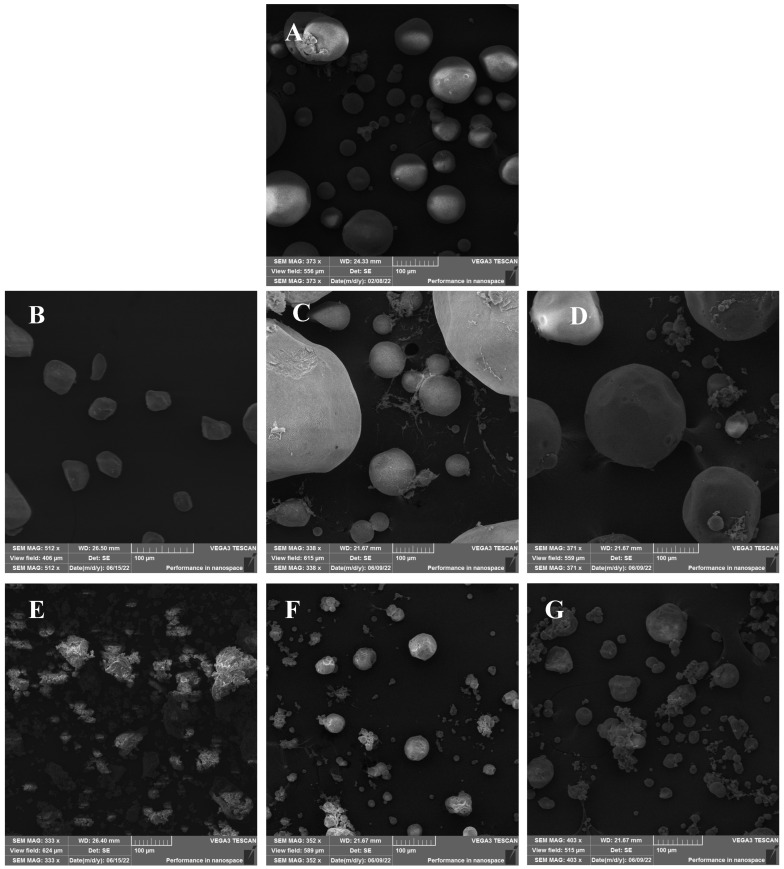
SEM images for (**A**) alginate, (**B**) pectin, (**C**) alginate/pectin 1:1 (AP1), (**D**) alginate/pectin 2:1 (AP2), (**E**) carrageenan, (**F**) alginate/carrageenan 1:1 (AC1), and (**G**) alginate/carrageenan 2:1 (AC2).

The SEM images for alginate showed spherical-like microparticles of different sizes spanning 20–100 µm (Figure 1). Pectin aerogels exhibited irregular morphologies with smaller sizes (20–60 µm), while carrageenan aerogels displayed aggregated, coral-like structures with irregular particle sizes ranging from 20 to 120 µm. Among the hybrids, AP1 and AP2 produced large spherical particles (80–400 µm), whereas AC1 and AC2 exhibited more defined particle shapes compared to carrageenan aerogel with particle sizes between 20 and 140 µm. These morphological differences may be attributed to variations in sol viscosity and gelation kinetics among the biopolymers. Hybridization improved the particles’ shapes, especially in the AP2 and AC2 aerogels, which exhibited a more defined spherical structure and reduced aggregation tendency. This suggests that alginate plays a dominant structural role in hybrid networks and potentially acts as a matrix backbone. Although laser diffraction measurements were not performed to obtain full particle size distributions, image-based analysis confirmed that the aerogels exhibited particle sizes comparable to values reported in the literature for polysaccharide-based aerogel microparticles [17]. Future studies should apply dynamic particle sizing techniques to provide a more detailed characterization.

The BET surface area and the BJH-derived pore size and pore volume are presented in Table 1. A relatively similar surface area for alginate aerogel microparticles was produced compared with similar works in the literature (330–680 m^2^/g). [17,34,38]. The variation in the textural properties of the produced aerogels is related to the differences in the alginate source and batch, the gelation process, and the effectiveness of the drying process [36,39]. The BET and BJH analyses supported the morphological findings, showing that hybrid aerogels achieved enhanced surface area and pore volume relative to single pectin and carrageenan aerogels. Alginate-based aerogels have the highest surface area (521 m^2^/g), pore size (21.35 nm), and pore volume (3.4 cc/g) compared to the other two single-polymer aerogels (pectin and carrageenan); the polymer material incorporated in producing the aerogel affects the surface area, pore size, and pore volume. The surface area of the pectin-based aerogel was within the reported values in the literature, which varied from 270 to 600 m^2^/g [17,40,41]. This variability may be related to the pectin source, where the physical stability and specific surface area are influenced by the degree of esterification, which varies from one source to another. It could also be attributed to the variability in the gel’s crosslinking degree. Carrageenan-based aerogel displays the smallest surface area among the other tested biopolymers, indicating that the aerogel production process could be optimized. However, the values were comparable to those reported by Obaidat et al.

Hybrid alginate/pectin aerogels showed a higher surface area than single pectin polymer aerogels and smaller than that of alginate. In addition, alginate/pectin aerogels with higher alginate content (sample AP2) presented the highest surface area of all measured hybrid samples. The increase in surface area and porosity can be partly explained by the “egg-box” model of crosslinking, where divalent calcium ions interact with guluronic acid residues in alginate and carboxylic groups in pectin. The enhanced network structure in hybrid formulations indicates synergistic crosslinking. More investigations on the hybridization between alginate and pectin and their effect on each other are needed.

Similar behavior was observed for alginate/carrageenan aerogels, where increasing the alginate content in the gel matrix significantly increased the internal surface area. Here, it could be assumed that the physical crosslinking of alginate with Ca^2+^ ions is mainly responsible for gelation, and carrageenan mostly acts as a filler material that fills the voids in the gel matrix, considering that Ca^2+^ induces gelation in carrageenan, thereby increasing the internal surface area.

### 2.2. Thermogravimetric Analysis (TGA)

**Figure 2:**
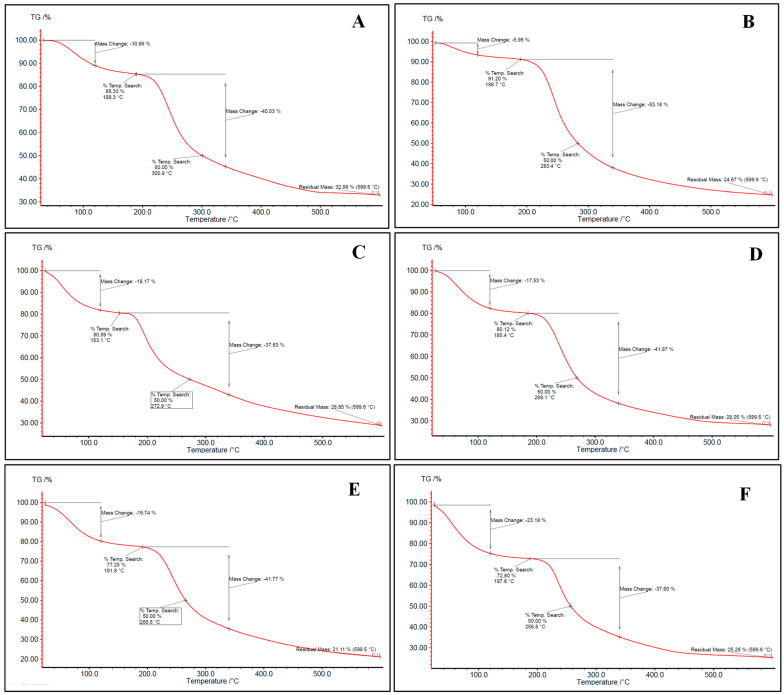
TGA thermogram for (**A**) alginate, (**B**) pectin, (**C**) carrageenan, (**D**) alginate/pectin 1:1, (**E**) alginate/pectin 2:1, and (**F**) alginate/carrageenan 2:1 aerogels.

Aerogels were evaluated for their thermal properties through thermogravimetric analysis (TGA). The TGA thermograms (Figure 2) of all aerogels exhibited three-stages degradation behavior. The first occurred at 50–150 °C, related to surface and inner moisture evaporation. The associated water loss at this stage was in the range of 5.95–26.51%. Similar results were found in the literature [12,42]. The second stage occurs in the range of 180–360 °C and corresponds to the depolymerization of the polymer network structure. Most of the weight loss of the samples upon heating refers to this stage, where 41.5% of the mass is lost. The last stage begins at approximately 360 °C for most bio-aerogels and continues until the aerogel is completely decomposed. This stage reflects the complete thermal decomposition of the polysaccharides, resulting in the formation of residual ash. Similar findings were also presented by [42].

Hybridization between alginate and other polysaccharides did not significantly change the thermal stability of alginate and pectin-based aerogels (Figure 2). Despite carrageenan having the lowest depolymerization temperature, alginate/carrageenan aerogels showed stabilization where decomposition temperature increased to reach the depolymerization temperature of alginate. This may reflect hydrogen bonding or entanglement, which could also explain the more consistent particle morphology in SEM images.

### 2.3. Differential Scanning Calorimetry (DSC)

**Figure 3:**
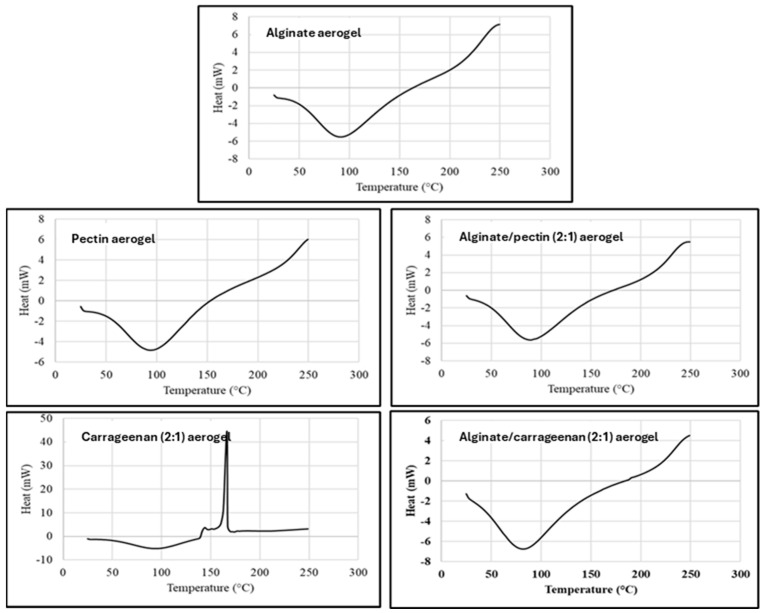
DSC thermogram for alginate, pectin, carrageenan, alginate/pectin (2:1), and alginate/carrageenan (2:1) aerogels.

DSC thermal analysis records the absorbed heat upon heating the material at a constant rate (10 °C/min), and DSC thermograms represent the thermal behaviors and phase transitions aligned with heat flow in the examined temperature range. Figure 3 shows the DSC thermograms for alginate, pectin, carrageenan, and hybrid aerogels, that are in agreement with TGA results and previous reports [42,43]. All evaluated aerogels exhibited the typical endothermic peak between 90 and 100 °C ascribed to the loss of bound water from polymers, as the affinity of these aerogels to water is expected from their nature as dry polysaccharide hydrophilic polymers.

The DSC thermogram for κ-carrageenan aerogel showed an exothermic peak at 166 °C, attributed to the thermal degradation or depolymerization of the polysaccharide. This confirms the result by TGA, where the depolymerization started at about 153 °C. Obaidat et al. reported a similar exothermic peak for carrageenan [12].

The DSC of the hybrid polymers shows thermograms similar to alginate and pectin aerogels. The characteristic degradation exothermic peak of carrageenan disappeared in the hybrid aerogels. This can indicate the thermal stabilization of the carrageenan polymer in hybridization with alginate. This result matches very well with the results obtained from the TGA thermogram.

### 2.4. Loading the Aerogels with Ibuprofen

#### 2.4.1. Loading Efficiency

Four different samples were selected for drug loading and dissolution tests namely alginate, pectin, alginate/pectin (1:1), and alginate/carrageenan (2:1). The samples were selected to have different textural characteristics in terms of specific surface area, pore size, and pore volume. Table 2 shows the loading percentage of ibuprofen in selected aerogels. Statistical significance was determined by one-way ANOVA with Tukey’s post hoc test; different superscript letters within a column indicate significant differences (*p* < 0.05).

The loading efficiency of AC2 aerogels was the highest, followed by ALG, while PEC and AP3 showed the lowest efficiencies. Statistical analysis confirmed significant differences between PEC and the hybrid formulations (*p* < 0.05), whereas ALG and AC2 did not differ significantly, indicating comparable encapsulation performance. The lower loading efficiency of PEC and AP3 can be attributed to their reduced surface area and pore volume compared with other aerogels, which limited drug entrapment. In contrast, the high surface area of ALG/κ-carrageenan (2:1) and the large pore diameter of ALG contributed to their superior loading capacities. Similar trends have been reported for alginate cryogels, where increased porosity is directly associated with improved drug loading [44].

The surface chemistry of polysaccharide aerogels strongly influences drug loading, as functional groups such as carboxylates (alginate, pectin) and sulfates (κ-carrageenan) carry negative charges under physiological pH. These charges promote hydrogen bonding and electrostatic interactions with ibuprofen, consistent with the shifts observed in the FTIR spectra. Although zeta potential was not determined in this study, the literature reports confirm that alginate, pectin, and carrageenan aerogels exhibit negative surface charges due to their anionic functional groups [12,17]. Future studies will include zeta potential measurements to provide a more quantitative understanding of surface charge and its correlation with encapsulation efficiency.

While ibuprofen (Class II) exemplifies hydrophobic drugs well suited to scCO_2_ impregnation (adsorption–precipitation within mesopores), hydrophilic drugs are better addressed via post-loading in aqueous media (diffusion into the gel network) and by ionic/affinity interactions with the matrix (e.g., carboxylate groups in alginate/pectin; sulfate groups in κ-carrageenan) to avoid rapid wash-out. Co-solvent exchange (water/ethanol) can also assist the loading of amphiphilic or moderately hydrophilic drugs. These strategies have been widely reported for biopolymer aerogels and enable formulation-specific tuning of retention and release profiles [45].

#### 2.4.2. Ibuprofen In Vitro Release Study

**Figure 4:**
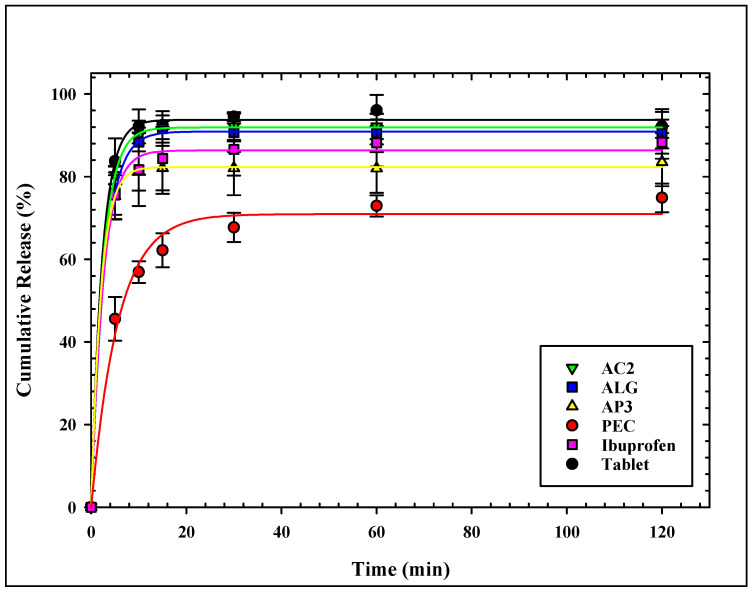
Cumulative release profile of ibuprofen from all loaded aerogels.

The dissolution profiles of ibuprofen from all loaded aerogels are presented in Figure 4. All aerogels showed similar release patterns of ibuprofen, with slight differences at the initial release.

ALG and AC2 showed the fastest and highest release profiles of all aerogels. More than 75% of ibuprofen was released from the ALG sample, the aerogels within 5 min, and more than 90% were released within 15 min. The same attributes were observed for the AC2 sample. The release profile of ALG and AC2 match well with the release profile of Ibuprofen^®^ tablets. On the other hand, pectin-based aerogels showed the slowest ibuprofen release rate with the lowest release percentage over the test period, not exceeding 75% after 120 min. Hybridization with alginate (AP3) showed improved release for ibuprofen, where more than 75% release was achieved within the first 5 min. It is noticed that alginate carrageenan (AC2) shows a faster release profile compared to alginate pectin (AP3) or any other tested aerogel formulation.

The single-polymer aerogels (ALG, PEC, and CAR) were included in this study as controls, providing baseline performance values for comparison with binary hybrids. As expected, the controls confirmed the intrinsic behavior of each polymer: ALG supported fast release and high loading efficiency, PEC showed slower release and lower loading, and CAR displayed intermediate performance. The hybrid formulations (AP, AC series) were then benchmarked against these controls, which allowed us to quantify the contribution of hybridization to improved structural and functional performance.

A direct comparison between hybrid and single-polymer aerogels revealed significant differences in performance. For example, AC2 achieved the highest loading efficiency (93.5%) compared with ALG (83.5%) and PEC (58.0%) (see Table 2), while its cumulative release exceeded 90% within 15 min, closely tracking the reference tablet. In contrast, PEC released <75% at 120 min. These data indicate that alginate serves as a structural backbone that, when combined with κ-carrageenan, enhances matrix hydration and pore accessibility, thereby accelerating release; when combined with pectin, it mitigates slower PEC release through increased surface area and pore volume (Table 1). These observations are consistent with prior reports on hybrid alginate-based aerogels that demonstrated improved loading and tuned release behavior [2,34].

The burst release in the first 5 min is justified by the release of the loaded drug from the outer surface of the microparticles and a rapid collapse of the aerogel structure.

ALG and AC2 had the maximum pore diameter that enhanced rapid ibuprofen release, where the release from the two aerogels did not show a significant difference from the ibuprofen tablet. The same observation on the enhancement of the release profile of ketoprofen from alginate and hybrid alginate/carrageenan over alginate/pectin was found by Gonçalves et al. [34].

Pectin-based aerogels showed the slowest ibuprofen release with the lowest release percentage over the test period. García-González et al. [17] explained the incomplete release of ketoprofen from pectin aerogels compared to alginate to the lower surface area, the larger particle size of pectin, and drug–matrix interactions. The relatively low average pore volume of the pectin-based aerogels limited the medium diffusion into the matrix. It hindered ibuprofen release desorption from the inner surface into the release medium, showing the least release. The enhancement of surface area by hybridization between alginate and pectin (sample AP2) is reflected in the enhanced release profile.

The similarity factor f2, which measures the closeness between the release profile of ibuprofen from the loaded aerogels and from the tablet, was calculated and listed in Table 2. An identical release profile contributes to f2 = 100, whereas a value of f2 = 50 is for an average variation of 10% at all time points. To ensure the sameness of the dissolution profiles, the FDA has established a public standard for f2 between 50 and 100 [46]. ALG has the highest similarity factor of all loaded aerogels, followed by AC2, whereas PEC and AP3 have a similarity factor less than 50.

Table 3 compares our results with selected alginate-based aerogels reported in the literature and with the commercial ibuprofen tablet. The data show that AC2 (ALG/κ-CAR 2:1) achieved superior loading efficiency (93.5%) and rapid release (>90% within 15 min), closely resembling the dissolution profile of the reference tablet. ALG also demonstrated high porosity and strong similarity to the tablet (f_2_ = 78.8). In contrast, PEC showed markedly lower loading and slower release. The literature data for alginate aerogels (e.g., [47,48,49,50]) confirm their high porosity and, in some cases, high loading, but often with slower or pH-dependent release. These comparisons demonstrate that binary hybridization, especially ALG/κ-CAR, provides synergistic improvements in both drug loading and release performance compared with single-polymer aerogels.

The release kinetics of ibuprofen from the loaded aerogels were analyzed using multiple models, including zero-order, first-order, Higuchi, and Korsmeyer–Peppas. Although the first-order model adequately described the cumulative release profiles (R^2^ = 0.878–0.998), it is primarily empirical and does not provide mechanistic insight. In contrast, the Korsmeyer–Peppas model provided equally high or superior correlations (R^2^ > 0.99) while also yielding mechanistic information. The obtained exponent values (n ≤ 0.45, Table 2) correspond to Fickian diffusion (case I diffusion), in which the molar flux is proportional to the concentration gradient, as defined by Fick’s law. This indicates that ibuprofen release from ALG, AC3, and AP3, was governed predominantly by diffusion, while PEC showed a deviation suggestive of anomalous transport due to stronger drug–matrix interactions. The Korsmeyer–Peppas model thus captures the simultaneous processes of water diffusion into the matrix, swelling, and partial dissolution of the polysaccharide network, making it more appropriate for the present aerogel systems. These findings are consistent with prior reports, where Obeidat et al. described ibuprofen release from carrageenan aerogels by the Korsmeyer–Peppas model [12], and García-González et al. reported Korsmeyer–Peppas and first-order release kinetics for ketoprofen from alginate and pectin aerogels [17].

Hypothetically, tuning the alginate–co-polymer ratio modulates network density, porosity, and drug–matrix interactions, which in turn controls release kinetics. Increasing alginate raises Ca^2+^ crosslink density and tortuosity, reducing effective pore throats and attenuating early time release. Increasing κ-carrageenan enhances matrix hydration and water uptake, promoting faster early time release. Raising pectin content, particularly at lower degrees of esterification, strengthens drug–matrix interactions and decreases porosity, yielding slower profiles and a shift toward anomalous transport. Consequently, intermediate ratios (e.g., ALG/κ-CAR ≈ 2:1) can deliver an optimal trade-off, high loading, and rapid yet controlled release, consistent with the performance hierarchy observed across formulations.

#### 2.4.3. Physiochemical Characterization (XRD and FTIR)

##### XRD Patterns

**Figure 5:**
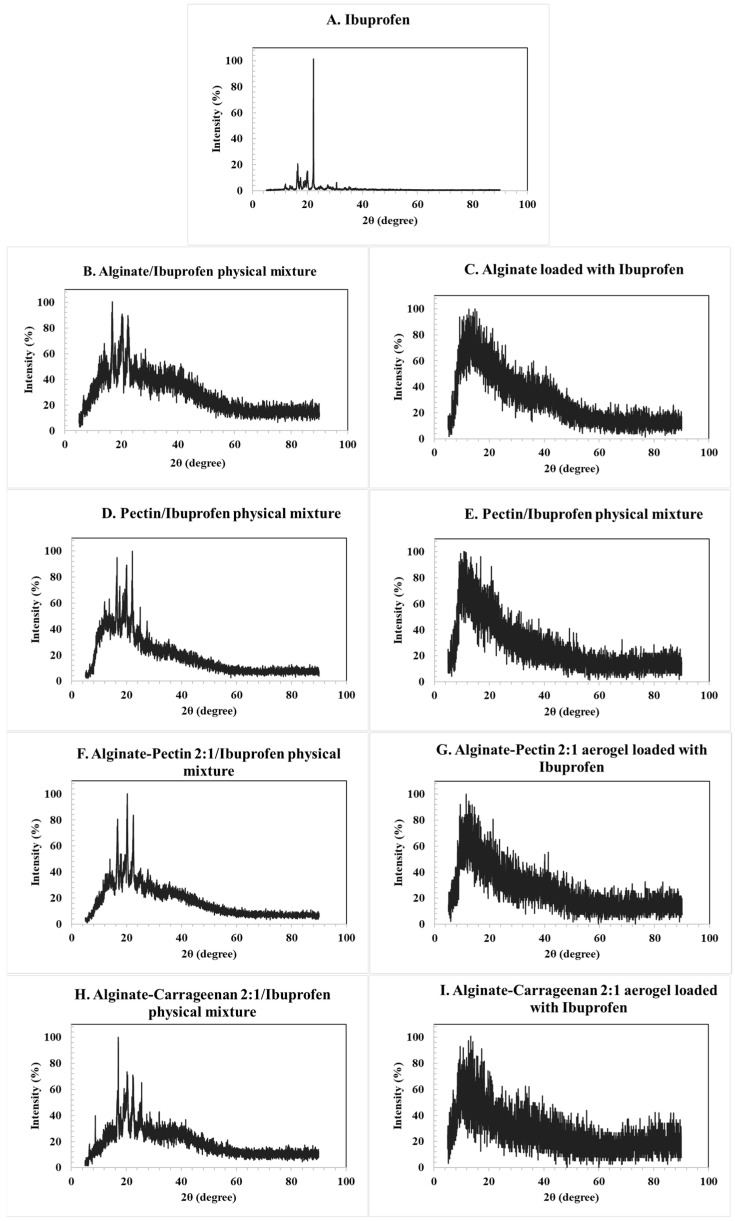
XRD pattern for the: (**A**) ibuprofen raw material, (**B**) corresponding alginate ibuprofen physical mixture, (**C**) alginate aerogel loaded with ibuprofen, (**D**) corresponding pectin ibuprofen physical mixture, (**E**) pectin aerogel loaded with ibuprofen, (**F**) alginate/pectin loaded with ibuprofen, (**H**) alginate/carrageenan loaded with ibuprofen, and their corresponding physical mixtures (**G,I**). Characteristic crystalline peaks of ibuprofen at 2θ ≈ 6.4°, 12.0°, 16.5°, 17.5°, 19.0°, 20.0°, and 22.4° are indicated in panel A, corresponding to the (002), (011), (110), and (111) planes. Peak positions were validated using structural reference data from the Crystallography Open Database (COD entries 2,006,278 and 2,104,678).

The XRD patterns of the loaded aerogels confirmed their amorphous character, as no distinctive crystalline peaks were observed for alginate, pectin, or their hybrids. In contrast, the diffractogram of pure ibuprofen (Figure 5A) exhibited sharp and intense reflections, consistent with its crystalline nature. The main characteristic peaks were observed at 2θ values of approximately 6.4°, 12.0°, 16.5°, 17.5°, 19.0°, 20.0°, and 22.4°, in agreement with reference data derived from the Crystallography Open Database (COD entries 2,006,278 and 2,104,678). These reflections correspond to the (002), (011), (110), and (111) planes of crystalline ibuprofen.

The physical mixtures of ibuprofen with alginate and pectin aerogels (Figure 5B,D) retained these crystalline peaks, as expected. However, in the drug-loaded aerogels (Figure 5C,E), the characteristic reflections disappeared and were replaced by a broad diffuse halo, indicating the absence of long-range order and the conversion of ibuprofen into an amorphous state. Similar behavior was observed for the hybrid alginate/pectin and alginate/carrageenan systems (Figure 5F,H). These results confirm that the drug was incorporated into the aerogel matrices in an amorphous form rather than in a crystalline state [12,51].

The drug-loading process occurred primarily through adsorption–precipitation from supercritical CO_2_. Under the selected conditions, ibuprofen dissolves in scCO_2_ and diffuses into the aerogel’s pore network. Upon depressurization, supersaturation occurs and the drug precipitates within the mesopores form amorphous deposits that maximize surface interaction with the carrier. This mechanism is consistent with the absence of sharp crystalline peaks and the appearance of broad diffuse scattering in the XRD patterns, indicating that the drug was entrapped within the aerogel matrix rather than simply deposited on the outer surface. FTIR spectra further revealed hydrogen-bonding interactions between the carboxyl groups of ibuprofen and the hydroxyl groups of the polysaccharides, supporting stabilization of the amorphous form [52,53].

##### FTIR Analysis

**Figure 6:**
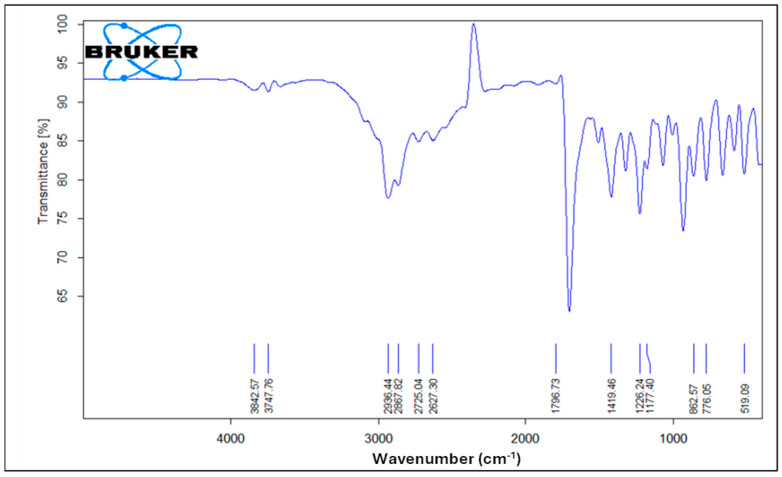
FTIR spectrum of pure ibuprofen.

Figure 6 illustrates the FTIR spectrum of pure ibuprofen. An intense and well-defined band at 1796 cm^−1^ attributed to the key functional group in ibuprofen’s structure is assigned for the carbonyl C=O stretching of the isopropionic acid group. A strong absorption band at 2936 cm^−1^ is assigned to CH_3_ stretching. A peak of strong intensity at 1226 cm^−1^ is due to C-C stretching. An O-H stretching in the hydroxyl group is also observed at 3842 cm^−1^. A band at 776 cm^−1^ is observed and related to CH_2_ vibrations. Ibuprofen fingerprint bands were assigned in the literature [12,54,55].

**Figure 7:**
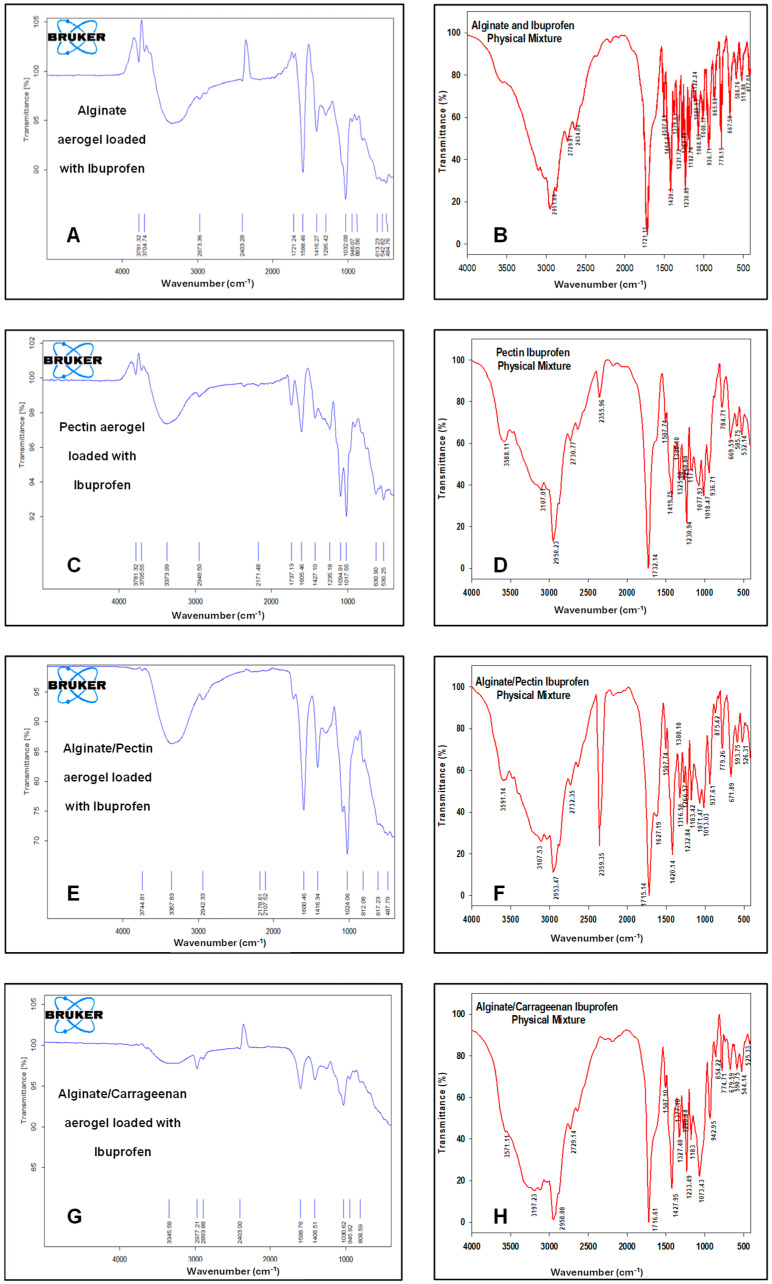
FTIR spectrum for: (**A**) alginate aerogel loaded with ibuprofen, (**B**) alginate aerogel/ibuprofen physical mixture, (**C**) pectin aerogel loaded with ibuprofen, (**D**) pectin aerogel/ibuprofen physical mixtures. (**E**) Alginate/pectin aerogel loaded with ibuprofen, (**F**) alginate/pectin aerogel with ibuprofen physical mixture, (**G**) alginate/carrageenan aerogel loaded with ibuprofen, and (**H**) alginate/carrageenan and ibuprofen physical mixtures.

Figure 7 illustrates the FTIR spectra of the drug-loaded aerogels along with their physical mixtures. The characteristic peaks of ibuprofen appeared in all FTIR spectra of loaded formulations and physical mixtures, respectively, confirming that ibuprofen was successfully loaded into these formulations. CH_3_ stretching at 2936 cm^−1^ was observed without any significant shifts. The carbonyl (C=O) stretching at 1796 cm^−1^ and C-C stretching at 1226 cm^−1^ appeared only in alginate and pectin aerogels and were absent in the alginate/pectin 2:1 and alginate/carrageenan 2:1 aerogels. However, the broad O-H stretching band at 3842 cm^−1^ shifted to about 3781 cm^-1^ in the alginate, pectin, and alginate/pectin 2:1 aerogels and was absent in the loaded alginate/carrageenan 2:1 aerogel. Hybrid alginate/pectin showed new peaks at about 1100 cm^−1^ and 1720 cm^−1^, indicating crosslinking of the prepared aerogel, indicative of C-O-C and COOH stretching vibrations, respectively. This confirms the results of [42]’s study. The characteristic peak of COO was shifted to a lower value of 1416 cm^−1^ in both the alginate/pectin aerogel and alginate/carrageenan aerogel, indicating crosslinking by hydrogen bonding in both aerogels. The other characteristic peaks of alginate and pectin remained at the same wavelength after hybridization.

It is worth mentioning that polysaccharide aerogels are generally stable under dry storage for extended periods, with reports indicating preservation of textural properties and structural integrity for >12 months. Drugs confined in aerogel mesopores often remain in the amorphous state due to nanoconfinement effects that suppress recrystallization [2,17,56]. Nonetheless, long-term stability studies under the guidelines of stability testing of pharmaceutical products will be necessary to confirm the shelf life and ensure regulatory compliance for pharmaceutical translation.

## 3. Conclusions

This presented study successfully demonstrated the production of hybrid polysaccharide-based aerogels using alginate, pectin, and carrageenan through emulsion–gelation followed by a stepwise solvent exchange and supercritical CO_2_ drying. The resulting aerogels exhibited tunable morphology with high specific surface area and enhanced thermal properties. Hybridization with alginate significantly improved the physical characteristics compared to single-polymer aerogels, particularly in terms of surface area, pore volume, and drug-loading capacity. Alginate played a dominant role in strengthening hybrid matrices and improving overall aerogel architecture. Drug loading and release studies reveal that the alginate/carrageenan (2:1) hybrid aerogel achieved the highest drug loading efficiency and exhibited rapid ibuprofen release kinetics comparable to that of commercial tablets. The drug release mechanism was governed by Fickian diffusion, indicating that the interconnected porous structure of hybrid aerogels facilitates efficient and fast drug transport. These findings highlight the potential of hybrid polysaccharide-based aerogels as a promising platform for drug delivery applications.

The processing steps used in this study, emulsion–gelation and supercritical CO_2_ drying, are amenable to scale-up and industrial translation. Emulsion–gelation is already employed in the food and pharmaceutical industries for producing microspheres and beads, and continuous setups have been developed to increase throughput. Similarly, ScCO_2_ drying has been commercialized for large-scale food processing (e.g., decaffeination) and drug impregnation, with well-established safety and GMP protocols. The combination of these processes enables reproducible production of aerogel microparticles at industrially relevant scales, supporting their future application in oral and pulmonary drug delivery [2,27,38].

More research should be conducted on optimization of hybridization ratios, crosslinking parameters, and the emulsion–gelation method to enable aerogel tailoring for specific applications. Additionally, the developed platform should be expanded to poorly soluble and control release drugs. Additionally, future research should include in vivo pharmacokinetic and pharmacodynamic studies to validate the performance of these systems beyond in vitro dissolution. For oral administration, rodent (rat) and rabbit models are commonly employed for evaluating bioavailability and systemic exposure. Moreover, extending this platform to hydrophilic model drugs will further test the versatility of hybrid aerogels. These steps will help translate the promising in vitro performance of hybrid alginate-based aerogels into preclinical applications.

By combining high drug-loading capacity, tunable release behavior, and biocompatibility, these hybrid aerogels provide a versatile platform for innovative biomedical applications, including oral, pulmonary, and topical drug delivery.

## 4. Materials and Methods

### 4.1. Materials

Sodium alginate was supplied from Carl Roth, Germany. Pectin and Span 80 were provided by TCI, Tokyo, Japan. κ-carrageenan was purchased from SIGMA, Søborg, Denmark. Calcium carbonate was provided by SIGMA-ALDRICH, Darmstadt, Germany. Paraffin oil was supplied by Al Yassur for technical and scientific supplies, Amman, Jordan. Glacial acetic acid was purchased from Scharlau, Barcelona, Spain. Carbon dioxide (CO_2_) with a purity of 99.995% was supplied from Rum for Gasses, Amman, Jordan. Absolute ethanol was provided by Solvochem, Rotterdam, Holland. Deionized water was used throughout the study. All materials were used as supplied without any further processing.

### 4.2. Methods

In this study, several processing parameters were evaluated, namely: biopolymer type, crosslinker concentration, hybridization, and biopolymer ratios. Table 4 summarizes the produced matrix, biopolymers used, their ratios, and the name assigned to each product. Biopolymeric aerogels were prepared by combining the internal setting method with the emulsion–gelation procedure [38]. The following sections thoroughly outline a detailed description of the aerogel preparation.

#### 4.2.1. Preparation of Single Polymer Gels Using the Emulsification Method

Several techniques are available for the preparation of alginate-based microparticles via diffusion-driven gelation, where alginate droplets are introduced into a solution containing divalent cations such as Ca^2+^ and undergo ionic crosslinking. These include the traditional dripping method and variations in the original technique in which electrostatic, mechanical, or vibrational forces are applied to break the droplets prior to gelation [57]. The jet cutting technique has also been reported, in which mechanical forces act on a fluid jet to produce particles with controlled sizes, typically above 100 µm [58]. While these approaches are suitable for producing millimeter- to sub-millimeter-sized beads, for internal setting methods, the emulsion–gelation process is the most effective strategy for generating homogeneous microparticles. In addition to ensuring particle uniformity, emulsion–gelation provides reproducible control over particle morphology, facilitates large-scale production, and has been successfully applied to prepare polysaccharide-based aerogels for drug delivery in prior studies [57,59,60].

Aqueous hydrogel phases were prepared using alginate, pectin, and carrageenan polymers as follows: accurate amounts of each polymer were mixed with water to produce total polymeric concentrations of 3%, 3%, and 1% for alginate, pectin, and κ-carrageenan, respectively. This mixture was mixed under magnetic stirring with water for 3 h and left overnight for complete hydration. Then, calcium carbonate at a ratio of 0.1825 g CaCO_3_/g biopolymer was added and mixed just before emulsification. The oil phase was prepared by mixing Span 80 with paraffin oil to achieve a 1% *w*/*w* concentration.

The emulsification step was achieved by adding the oil phase to the aqueous polymeric phase with a ratio of 3:1, with continuous mixing at 900 rpm for 1 h using an overhead mixer. Afterwards, the mixing speed was reduced to 200 rpm, and for each 1 g of CaCO_3_, 2.4 g of acetic acid was added to the emulsion and mixed for a further 1 h. The mixture was left at 4 °C overnight for aging; then, the oil phase was removed, and the aqueous phase was collected by centrifugation at 4000 rpm for 15 min. Although droplet size distribution was not directly measured, SEM analysis indicated microparticles ranging from 20 to 100 µm for alginate-based aerogels. Future studies will include direct droplet size analysis to further validate size uniformity.

#### 4.2.2. Preparation of Hybrids of Alginate/Biopolymer Gels

Hybrid hydrogels were prepared by mixing the targeted ratios of alginate/biopolymer of 1:1 or 2:1, then adding CaCO_3_. The amount of CaCO_3_ used to prepare hybrid alginate/pectin hydrogels was 0.1825 g CaCO_3_/g alginate, and two different quantities of CaCO_3_ concerning pectin: 0.0913 g CaCO_3_/g pectin (q = 1) or 0.1825 g CaCO_3_/g pectin (q = 2). Then, the emulsification process was used, as mentioned earlier.

In this study, binary alginate-based hybrids (ALG/PEC and ALG/CAR) were selected to systematically assess the complementary effects of pectin and carrageenan on alginate aerogels. Pectin provides additional carboxyl groups that can retard drug release, whereas carrageenan introduces sulfate groups that enhance water uptake and accelerate matrix hydration. Evaluating binary hybrids enabled clear attribution of observed effects to one secondary polysaccharide. Ternary blends (ALG/PEC/CAR) were not investigated here due to their more complex gelation kinetics and higher risk of phase separation, but they present a promising direction for future work.

#### 4.2.3. Solvent Exchange

A successive stepwise solvent exchange by ethanol–water mixtures of 30, 60, 90, 100, and 100% *v*/*v* was performed to replace the water in the collected gel particles with ethanol before introducing them to scCO_2_ drying. Each step includes 30 min of mixing and 15 min of centrifugation at 4000 rpm. A repeated solvent exchange step with 100% ethanol was used to ensure that the ethanol concentration in the gel microparticles is higher than 98%.

#### 4.2.4. Supercritical Drying

Supercritical CO_2_ drying was followed as described by [2,17,61]. The collected alcogel particles are packed into a bag-filter paper and placed in a 0.5 L stainless steel vessel, previously heated to 40 °C. ScCO_2_ was continuously driven to the vessel at a rate of 80–120 g/min by a pneumatic-driven pump. A back-pressure regulator was used to control the pressure inside the vessel and maintain it at 120 bars during drying. The ethanol–CO_2_ phase was passed through a separation vessel at 60 bars and 40 °C, where ethanol was collected from the bottom, and CO_2_ was recycled to the extraction process. Fresh CO_2_ was continuously supplied to the extraction process to guarantee sufficient extraction. The extraction lasted for 6 h to ensure complete drying. Finally, the pressure inside the vessel was reduced slowly to ambient conditions. Using a unified protocol across all formulations enabled direct comparison of single- and hybrid-polymer aerogels. Although formulation-specific optimization could be pursued in future work, the selected conditions represent a well-established standard for biopolymer aerogels.

#### 4.2.5. Drug Loading

In the presented work, ibuprofen as a model drug was loaded into the aerogel matrix. The loading was performed by impregnation of the aerogel in scCO_2_. About 0.8 g of aerogel particles and 0.2 g of ibuprofen were weighed in separate cartridges of bag filter papers, and placed in a preheated high-pressure vessel at 40 °C. After that, the vessel was pressurized to 100 bars using a pneumatic-driven pump. The condition was maintained for 2 h [12]. The target ibuprofen loading percentage was 20%. The loaded aerogel particles were collected from the autoclave after depressurizing the system and preserved in a desiccator under a humidity-free atmosphere for further processing.

### 4.3. Physicochemical Characterizations of Aerogel Aerogels

#### 4.3.1. Scanning Electron Microscopy (SEM)

TEscan VEGA3, Brno, Czech Republic, was used to study the aerogels’ textural properties and surface morphology at a working distance of 22 mm and beam energy of 10 kV. Before the analysis, all samples were placed on stubs and coated with gold under a vacuum atmosphere using a Q150R Rotary-Pumped Sputter Coater/Carbon Coater (Quorum Technologies, Kent, UK).

#### 4.3.2. Specific Surface Area and Porosity

The Brunauer–Emmet–Teller (BET) and Barrett–Joyner–Halenda (BJH) methods were used to determine the specific surface area and pore volume using low-temperature nitrogen adsorption–desorption analysis by NOVA 2000 series (QUANTACHROME, Boynton Beach, FL, USA). Each sample was degassed at 55 °C for 24 h before measurement.

#### 4.3.3. Thermogravimetric Analysis (TGA)

Thermogravimetric TGA analyses were carried out using the TG209 F1 Libra (Wittelsbacherstraße 42, Selb, Germany). An accurately weighed sample was heated from 25 to 600 °C at a rate of 10 °C/min under nitrogen purge and a protective flow of 20 mL/min. The original mass of the sample was used to calculate the mass loss percentage T50%, and the residue after 600 °C.

#### 4.3.4. Differential Scanning Calorimetry (DSC)

DSC thermograms of the polysaccharide-based aerogels were obtained using DSC DSC823e (METTLER TOLEDO, Greifensee, Switzerland). About 5 mg of each sample was heated from 25 to 250 °C at a rate of 10 °C/min under nitrogen using an empty sealed aluminum pan as a reference.

### 4.4. Loading of the Prepared Aerogels with Ibuprofen

#### Loading Efficiency

A total of 50 mL of phosphate buffer (pH 7.2) was added to accurately weighed loaded aerogels containing 5 mg of drug in a 100 mL flask. Sonication was performed for 5 min. Then, phosphate buffer was added so the volume reached 100 mL, followed by sonication for an extra 5 min. A sample of this mixture was filtered, and its absorbance was recorded by a UV spectrometer at 221 nm. The loading efficiency for each drug loaded aerogel was evaluated using Equation (1).

(1) $$Loading\;Efficiency=\frac{Practical\;value}{Theoritical\;value}\times 100\%$$

### 4.5. Physicochemical Characterization of Drug-Loaded Aerogels

#### 4.5.1. Drug Dissolution Study

The in vitro dissolution study was carried out following the US Pharmacopeia with some modifications using a Dissolution Tester DIS 600i COPLEY, Nottingham, United Kingdom. The dissolution vessels were filled with 900 mL of the phosphate buffer (pH 7.2) and maintained at 37 °C in the test assembly. A total of 100 mg of each sample of the loaded aerogel particles was introduced into the dissolution vessels, and the medium was stirred at 50 rpm. A volume of 10 mL from each sample was withdrawn at time intervals of 5, 10, 15, 30, 60, 120, 180, and 240 min, and an equal volume from the fresh medium was replaced into the dissolution vessel after each sampling. The UV absorbance determined the amount of drug dissolved at 221 nm in the withdrawn portions. The cumulative percentage of drugs released from the aerogel particles was calculated and plotted as a function of time.

Release profiles are evaluated using fitting to the Korsmeyer–Peppas model, Equation (2) [62].

(2) $$\frac{Mt}{M\infty }={kt}^{n}$$

where $\frac{Mt}{M\infty }$ is the fraction of the drug released at the time (*t*), *k* is the rate constant, and *n* is the release exponent indicating the mechanism of release.

#### 4.5.2. XRD Patterns

X-ray diffraction patterns were carried out using a BRUKER D2 PHASER (Bruker AXS GmbH, Karlsruhe, Germany) at room temperature with diffraction angles from 5 to 90° of 2θ, at a voltage of 30 kV and a current of 10 mA. The step scan mode was used with a step size of 0.02°. The characteristic peaks of crystalline ibuprofen were identified by comparison with theoretical diffraction data derived from the Crystallography Open Database (COD entries 2,006,278 and 2,104,678). Assignments of major reflections (002), (011), (110), and (111) were used to confirm peak positions in the reference material. Rietveld refinement was not performed, as the objective was to confirm amorphization of the drug in the aerogel matrices rather than to carry out full quantitative phase analysis.

#### 4.5.3. Fourier Transform Infrared (FTIR) Spectra

The FTIR of the loaded aerogel and a physical mixture of raw drug and blank aerogel were analyzed using an IR Affinity-1 Spectrophotometer (Shimadzu, Kyoto, Japan). The spectra were measured in a range of (400–4000 cm^−1^) at room temperature, and the resolution was about 4.0 cm^−1^, using KBr pellets, Springfield, IL, USA.

## Figures and Tables

**Table 1:** Specific surface area, pore size, and pore volume of the studied samples.

Sample Name	Surface Area (m^2^/g)	Pore Size (nm)	Pore Volume (cm^3^/g)
ALG	521	21.35	3.40
PEC	321	12.41	2.01
CAR	233	13.90	1.99
AP1	365	17.41	2.31
AP2	431	22.50	2.81
AP3	356	13.65	1.85
AP4	413	16.32	2.55
AC1	315	14.24	2.98
AC2	385	17.24	3.52

**Table 2:** Loading percentage of ibuprofen in selected aerogels, similarity factor, and Korsmeyer–Peppas models fitting values of the selected aerogels.

Sample	Ibuprofen Loading %	Loading Efficiency	f_2_	Korsmeyer–Peppas	
				R^2^	n Value
ALG	16.7 ± 1.5	83.5 ^a^	78.8	0.996	0.025
PEC	11.6 ± 1.7	58.0 ^b^	23.2	0.993	0.108
AP3	12.3 ± 1.8	61.5 ^b^	43.6	0.999	0.023
AC2	18.7 ± 1.1	93.5 ^a^	64.9	0.996	0.030

Superscript letters denote statistical groupings (ANOVA, Tukey’s test, *p* < 0.05). Values sharing at least one common letter are not significantly different. Values with completely different letters are significantly different.

**Table 3:** Comparison of aerogel systems (surface area, loading, release, similarity factor).

System	Surface Area (m^2^/g)	Loading Efficiency (%)	% Release @ 15 min	f_2_ vs. Tablet	Notes
ALG (this work)	521	83.5	>90	78.8	High porosity, fast release
PEC (this work)	321	58.0	<75 (120 min)	23.2	Low porosity, slow release
AC2 (ALG/κ-CAR 2:1, this work)	385	93.5	>90	64.9	Synergistic hybrid, tablet-like release
AP3 (ALG/PEC 3:1, this work)	356	61.5	~85	55.3	Hybrid improves PEC performance
Alginate Aerogel Particles	~512	Up to ~30 (various drugs, including ketoprofen)	~70 (estimated from fast release vs. crystalline)	–	High porosity, amorphous stable 6 months; [47]
Fe(III)-Alginate Aerogel Beads	~316–442	36–41 (ibuprofen)	~30–50 (pH 7.4 faster than 2.0; estimated, accelerated with ascorbic)	–	Redox-responsive; high porosity; [48]
Bimetallic Alginate (Ca^2+^/Ba^2+^, Ibuprofen)	Not reported (Porosity: 58–79%)	Up to 95 (ibuprofen)	~40–50 (intestinal pH 7.2, from 96.9% in 1 h)	–	pH-responsive, fast intestinal release; [49]
Alginate Aerogels (Enteric Coated)	Not reported (>200 implied)	High (ibuprofen, reviewed)	<10 (no release pH 1.2, starts pH 7.4)	–	enteric coating via Wurster; porosity >90% [50]
Commercial Ibuprofen Tablet	N/A	N/A	>85 (reference profile)	–	Benchmark for dissolution performance

**Table 4:** Samples matrix used in this study.

Sample Name	Biopolymer/s Type	Biopolymeric Ratio
ALG	Alginate	100%
PEC	Pectin	100%
CAR	Carrageenan	100%
AP1	Alginate/pectin (q = 1) 0.0913 g CaCO_3_/g pectin	1:1
AP2		2:1
AP3	Alginate/pectin (q = 2) 0.1825 g CaCO_3_/g pectin	1:1
AP4		2:1
AC1	Alginate/carrageenan	1:1
AC2		2:1

## Data Availability

The original findings presented in this work are fully included in the article. Further details may be obtained by contacting the corresponding author.
